# Experimental Study of the Tensile Behavior of Structures Obtained by FDM 3D Printing Process

**DOI:** 10.3390/polym16111562

**Published:** 2024-05-31

**Authors:** Salem Ben hadj Hassine, Sami Chatti, Borhen Louhichi, Abdennour Seibi

**Affiliations:** 1 LMS, ISSATSo, University of Sousse, Sousse 4000, Tunisia; salem.haj007@gmail.com (S.B.h.H.); s.chatti.issat@gmail.com (S.C.); 2 Department of Mechanical Engineering, College of Engineering, Imam Mohammad Ibn Saud Islamic University (IMSIU), Riyadh 11432, Saudi Arabia; 3 Department of Engineering, Utah Valley University, 800 W University Pkwy, Orem, UT 84058, USA; aseibi@uvu.edu

**Keywords:** additive manufacturing, fused deposition modeling, 3D printing, process optimization, DOE, tensile properties, PLA

## Abstract

Fused Deposition Modelling (FDM) is one of the layer-based technologies that fall under the umbrella term “Additive Manufacturing”, where the desired part is created through the successive layer-by-layer addition process with high accuracy using computer-aided design data. Additive manufacturing technology, or as it is commonly known, 3D (three-dimensional) printing, is a rapidly growing sector of manufacturing that is incorporated in automotive, aerospace, biomedical, and many other fields. This work explores the impact of the Additive Manufacturing process on the mechanical proprieties of the fabricated part. To conduct this study, the 3D printed tensile specimens are designed according to the ASTM D638 standards and printed from a digital template file using the FDM 3D printer Raise3D N2. The material chosen for this 3D printing parameter optimization is Polylactic acid (PLA). The FDM process parameters that were studied in this work are the infill pattern, the infill density, and the infill cell orientation. These factors’ effects on the tensile behavior of printed parts were analyzed by the design of experiments method, using the statistical software MINITAB2020.

## 1. Introduction

Additive manufacturing (AM) or 3D printing is one of the leading technologies of rapid prototyping methods that creates a part from a virtual 3D CAD (Computer-aided Design) model to a real-life product. This technology has been integrated into a multitude of fields ranging from the automotive industry and the aerospace industry to the medical, architectural, and electronics sectors [1]. In modern manufacturing industries, due to the increased need for parts with great geometric complexity and relatively low weight, the use of material extrusion 3D printing has exponentially increased throughout the years to become an important technology [2]. The main appeal of AM, in contrast to the traditional manufacturing methods, is the possibility to create parts with complex geometry and complex internal channels, the creation of parts that are hollowed with lower weights which consequently increases the strength-to-weight ratio and significantly reduces the raw material used and production time [3].

**Figure 1:**
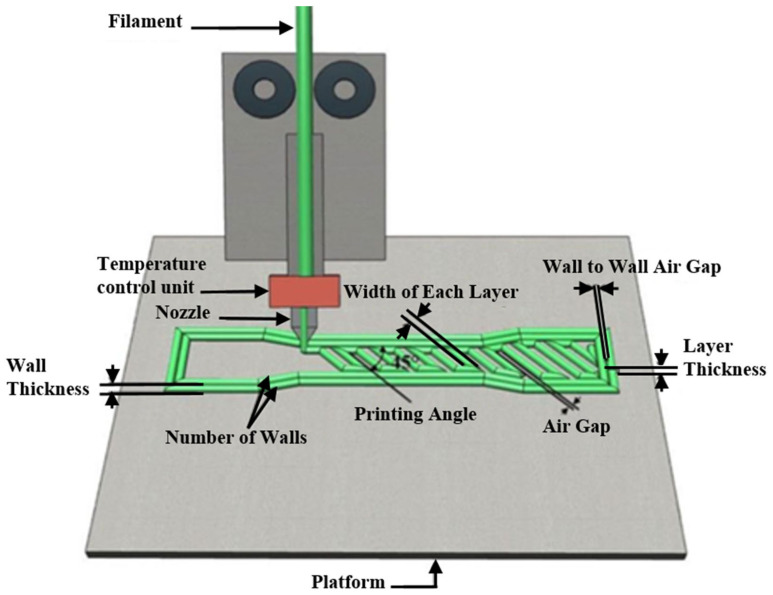
The interaction pathways between the physical and virtual models in the digital twin framework for additive manufacturing.

The AM technology comprises seven main methods that use the same principle, which include Vat photopolymerization, Material jetting, Binder jetting, Powder bed fusion, Fused deposition modeling, Sheet lamination, and Directed energy deposition [4]. Out of all these methods of 3D printing, fused deposition modeling (FDM) is the most heavily used one. The material is melted and then extruded in a pattern next to or on top of previous extrusions, layer by layer, to create a physical part. The material used in FDM printing is a thermoplastic polymer such as Polycarbonates (PC), polylactic acid (PLA), acrylonitrile butadiene styrene (ABS), nylon, Thermoplastic Polyurethane (TPU), acrylonitrile butadiene styrene (ABS) and many others [5]. The main reason for FDM printing becoming the most common method of additive manufacturing is due to its relative simplicity and the low cost of the printer and printing material. Parts that are produced with FDM are still a step behind in the mechanical properties compared to the traditional manufacturing methods and even other AM technologies, that is why it is paramount to identify and optimize its process parameters to create strong and reliable parts that can compete and provide a proper substitution to other parts created with other methods. Several process parameters can directly affect a part’s mechanical property, and these parameters are shown in Figure 1.

Numerous studies have been conducted to study the effect of these process parameters on the final properties of an FDM printed part and to optimize them [6]. The mechanical properties of a 3D printed part are the most important because they determine the real-life application and life span of the part. A wide range of optimization methods have been used to optimize the FDM process. Montero et al. [7] utilized a fractional factorial design to analyze five process parameters (raster width, air gap, raster orientation, filament color, and extrusion temperature). Their ABS-printed specimens were created on a Stratasys FDM 1650 unit (© Stratasys 2024). The results revealed that the raster orientation and the air gap were two important parameters for tensile strength, with a negative air gap and 0° raster orientation preferred to achieve an optimum tensile strength. Es-Said et al. [8] investigated the tensile properties of ABS parts generated by the FDM process using raster orientation as a variable. Parts were built in five different raster orientations. It was revealed that a raster orientation of 0° was the best for maximizing tensile strength. Alafaghani et al. [9] studied the effect of build orientation, infill density, infill patterns, print speed, extrusion temperatures, and layer thickness. They found that build orientation, layer thickness, infill density, and extrusion temperature were all significant for tensile properties (Young’s modulus, tensile strength, and yield strength) among the six parameters studied. Liu et al. [10] investigated layer thickness, raster orientation, raster width, printing orientation, and air gap to find the best process parameters for tensile properties. The three parameters that were found important were layer thickness (high), build orientation (60°), and raster orientation (−45°/45°).

Meanwhile, Tontowi et al. [11] conducted a study to predict the effects of varying raster angles, extrusion temperature, and layer thickness on the tensile strength. Layer thickness had the greatest impact on tensile strength. Maloch et al. [12] study found that increasing extrusion temperature and layer thickness can enhance mechanical properties. Chadha et al. [13] studied the effect of bed temperature and primary layer thickness on the mechanical properties. They showed that the strength increased with the temperature up to a certain point, then decreased. Fernandez et al. [14] found out that layer thickness significantly impacts tensile strength in 3D printed parts with PLA material, with the best strength achieved at 60% infill, 220 °C printing temperature, and 0.1mm layer thickness. Giri et al. [15] and Ziemian et al. [16] studies revealed that layer thickness, printing orientation, and cooling rate affect tensile strength and printing time of PLA material, with thicker layers resulting in weaker parts. According to Rankouhi et al. [17], the mechanical properties of ABS material are significantly affected by both layer thickness and raster orientation. Chacon et al. [18], using PLA material, evaluated the effect of the build orientation, feed rate, and layer thickness on the tensile and bending properties. Results showed that these parameters have a big impact on the output. The effect of various infill patterns on the compressive strength of ABS material was examined by Iyibilgin et al. [19]. Their research found that the honeycomb infill pattern resulted in the highest modulus. Gebisa et al. [20] conducted a study on ULTEM 9085 polymeric material and found that raster orientation significantly impacts tensile strength, while build time and surface quality are crucial for optimal performance. Panda et al. [21] focused on the effect of build orientation, layer thickness, raster width, raster orientation, and air gap on tensile strength. They discovered that all parameters (except raster width) and parameter combinations had an impact on tensile strength. Alafaghani et al. [22] studied the effect of the six printing parameters: build orientation, infill density, infill patterns (linear, diamond and hexagonal), print speed, extrusion temperatures and layer thickness on the tensile property parameters (Young’s modulus, tensile strength, yield strength, and ductility) of a PLA-material. It was found that the building direction and the infill density were the most significant parameters. However, the mechanical properties remain steady starting from the extrusion temperature of 185 °C. Additionally, it was observed that the print speed and the tested infill patterns had a negligible effect on the mechanical properties. However, the nozzle diameter has an effect on the mechanical parameter, as investigated by Tamașag et al. [23]. They considered only the Cubic infill pattern (grid), and they added contours (covering walls) to the tensile specimens. An increase of the tensile strength with increasing nozzle diameter was observed. Czyzewski [24] considered nozzles with diameters of 0.2 mm, 0.4 mm, 0.8 mm and 1.2 mm. The specimens were produced with a layer height of 0.2 mm and full filling (100%). They found that the lowest mechanical properties were obtained with a nozzle diameter of 0.2 mm. For nozzles diameters of 0.4 mm and 0.8 mm, the mechanical properties are almost the same. However, for the nozzle diameter of 1.2 mm, a degradation of the mechanical properties explained by the lack of connections between layers was observed. Many recent studies addressed this topic from another perspective and demonstrated that the mechanical performance of 3D-printed parts using FDM can be improved when the thermoplastic filament is reinforced with short fibers or a second nozzle during the FDM process to reinforce thermoplastic material with continuous fibers [25,26,27].

Previous studies that focused on the effect of infill patterns printed their test specimens with contour walls and top and bottom surfaces. In this study, our objective is to investigate the effect of process parameters on the mechanical properties by purely considering specimens without contour walls or top and bottom surfaces that could influence the results. The FDM process parameters investigated in this study include the infill pattern (Honeycomb, Gyroid, Grid, and Triangle), the infill density (40%, 50%, and 60%), and the infill cell orientation (0°, 45°, 90°). The impact of these factors on the tensile behavior of printed components is analyzed using the design of experiments method. Regression equations have been developed using surface response methodology, enabling the prediction of PLA tensile properties.

## 2. Material and Methods

Polylactic acid (PLA) is a biodegradable thermoplastic material created from renewable biomass, most commonly made from corn and sugarcane. PLA has a low melting temperature and low shrinkage rate when compared with other thermoplastic filaments. It can be blended with a variety of materials such as wood, carbon fiber, metal and stone to create PLA composites.

**Figure 2:**
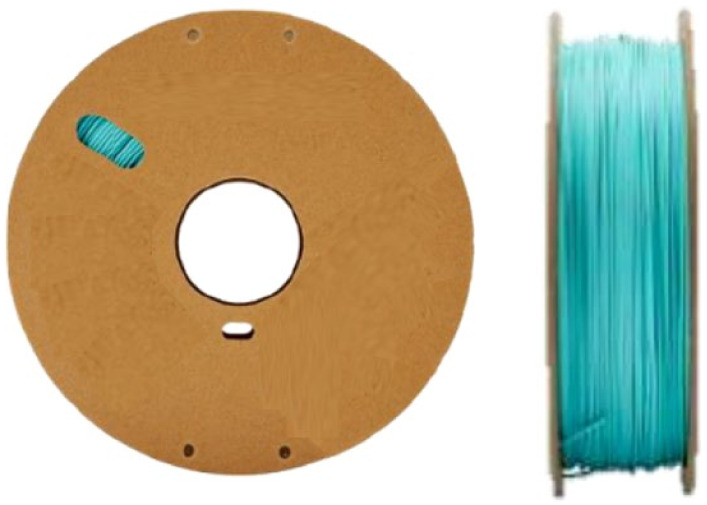
Polymaker polyterra PLA 1.75 mm/1 kg.

The material used in this work is a biodegradable polymer (polylactic acid PLA) derived from renewable resources, particularly starch extracted from corn, beets, and wheat [28]. The filament shown in Figure 2 has a diameter of 1.75 mm, a density of 1.31 g/cm^2^, tensile strength of 20.9 MPa, tensile modulus of 1882 Mpa and a charpy impact strength of 5.7 kj/m^2^. The filament has a 1.75 mm diameter. The filament is shown in Figure 2.

### 2.1. Specimen Design

**Figure 3:**
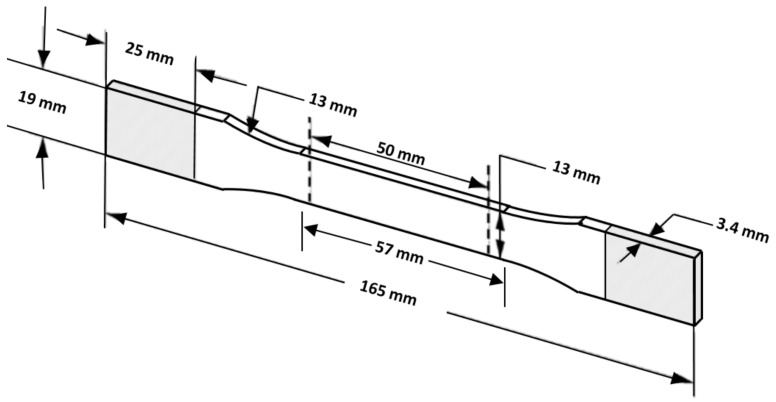
Tensile test specimen according to ASTM-D638 TYPE1 [29].

The tensile specimens, shown in Figure 3, are designed according to the ASTM-D638 type1 standard as used by Morettini et al. [29], among others. To produce a part using a 3D printer, its geometric specifications are necessary and are typically obtained from slicing software in the form of an STL file. In order for a 3D printer to create a part, it requires its geometric specifications that are obtained from a slicing software in the form of an STL file. IDEA MAKER slicing software (Version 4.3.0 Beta) was used because, unlike other slicers, it allows the printing of parts without walls or top and bottom surfaces in order to purely analyze the infill pattern. The slicer works by cutting the user’s model file into multiple layers, depending on the layer height, and generating a Gcode. Its main function is to instruct the printer how to move geometrically in 3 dimensions (x, y, z).

### 2.2. Printing Process

**Figure 4:**
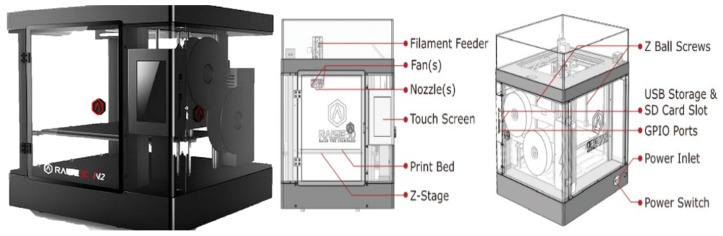
Raise3d N2 printer and components.

The PLA is heated and extruded through a nozzle to 3D print a cross-section of the specimen one layer at a time as per the Gcode instructions. With each layer, the bed is lowered automatically, and the process is repeated until the specimen is printed completely. Figure 4 depicts the 3D printer used in the experiment (Raise3DN2 - Raise 3D Technologies, Inc., Irvine, CA, USA). The printer’s technical specifications are:

- Print Technology: FDM.
- Build Volume (W × D × H): 305 × 305 × 305 mm.
- Layer Resolution: 0.01~0.25 mm.
- Compatible filament Type: PLA, ABS, PETG, Nylon.
- Filament Size: 1.75 mm.
- Printing Surface: Buildtak.
- Heated Build Platform: Yes.
- Enclosure: Yes.
- Nozzle Diameter: 0.4 mm.
- Nozzle Working Temperature: 170–300 °C.
- Number of Nozzles: 2.
- Printing Speed: 10~150 mm/s.
- Moving Speed: 150~300 mm/s.
- Positioning Accuracy: XY-axes: 0.0125 mm, Z-axis: 0.00125 mm.

Table 1 contains the default printing parameters considered for the subsequent experiments. As recommended by Zemcik et al. [30], the used layer height is equal to half of the Diameter nozzle.

### 2.3. Mixed Full Factorial Design of Experiments

The design of experiments (DOE) approach is a systematic method for determining the relation between parameters affecting the process’s output. It is utilized to discover cause-and-effect connections. The qualities of FDM printed parts are influenced by a variety of process parameters, and in this study, the parameters that were analyzed are the infill pattern, infill density, and infill cell orientation in order to determine their effect on the tensile behavior of the printed specimen. The infill pattern has four different levels while the other two factors have three different levels as shown in Table 2. A mixed full factorial design of experiments is defined by the selected three process parameters.

### 2.4. Tensile Testing

**Figure 5:**
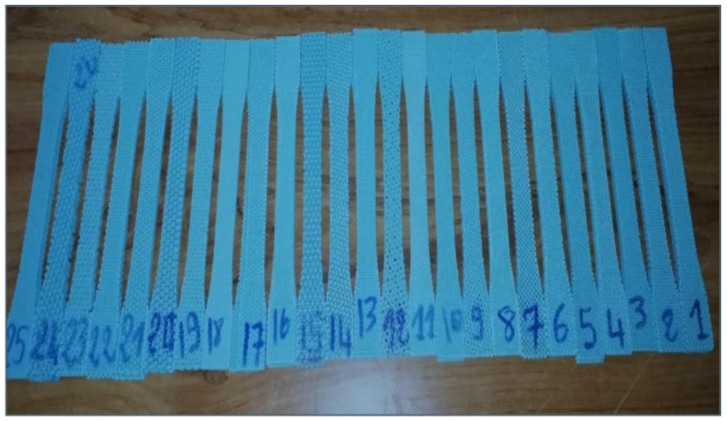
Some of the printed tensile specimens.

**Figure 6:**
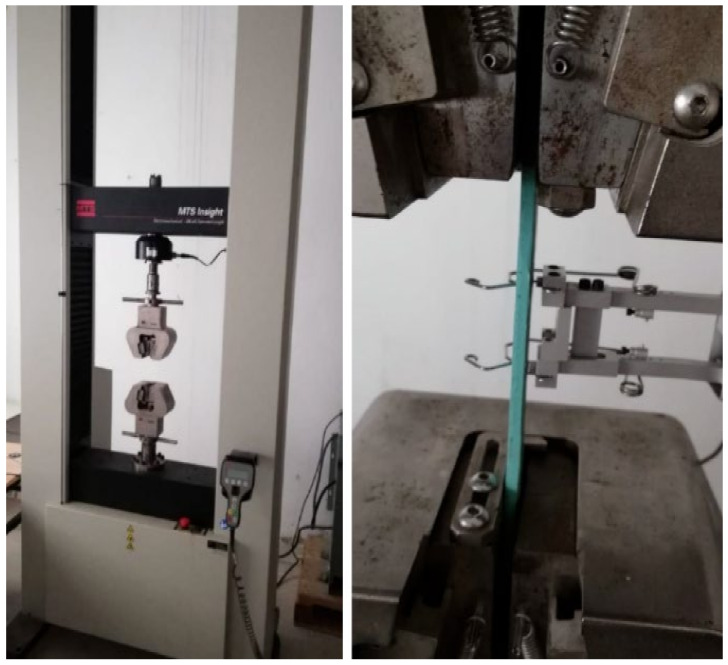
MTS insight tensile test machine with the specimen between the grips.

The printed specimens are shown in Figure 5 in accordance with ASTM D638 [29] standards. Tensile tests were executed on an MTS insight machine with an extensometer to accurately measure the displacement, as shown in Figure 6. It should be noted that each specimen was printed three times.

**Figure 7:**
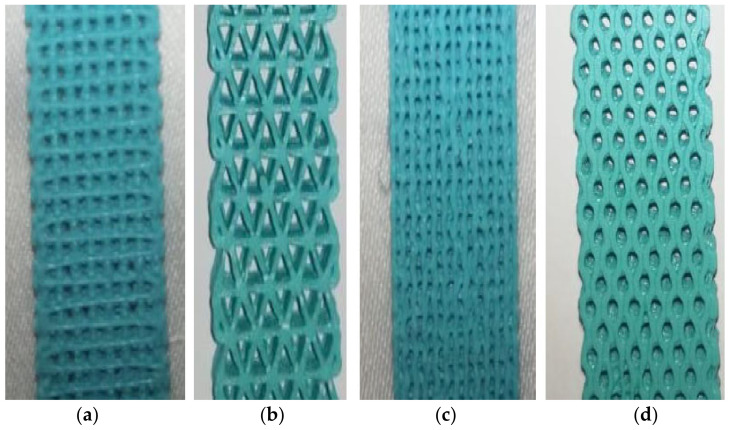
A close-up of some specimens with different infill patterns: (**a**) grid/(**b**) triangles/(**c**) gyroid/(**d**) honeycomb.

To properly see the internal structure of the tensile specimens, a close-up view is shown in the Figure 7 of several specimens with different infill patterns.

After conducting the tensile test, the yield strength (with 0.2% offset) and the elastic modulus were extracted from the tensile curve of each specimen and reported in Table 3.

## 3. Results and Analysis

DOE is a versatile data collection and analysis tool that can be used in a variety of studies. It enables the manipulation of a large number of input variables to determine their influence on the desired results (responses). By changing many inputs at the same time, DOE can uncover critical interactions that might otherwise go undetected when testing with one component at a time.

### 3.1. Pareto Charts

**Figure 8:**
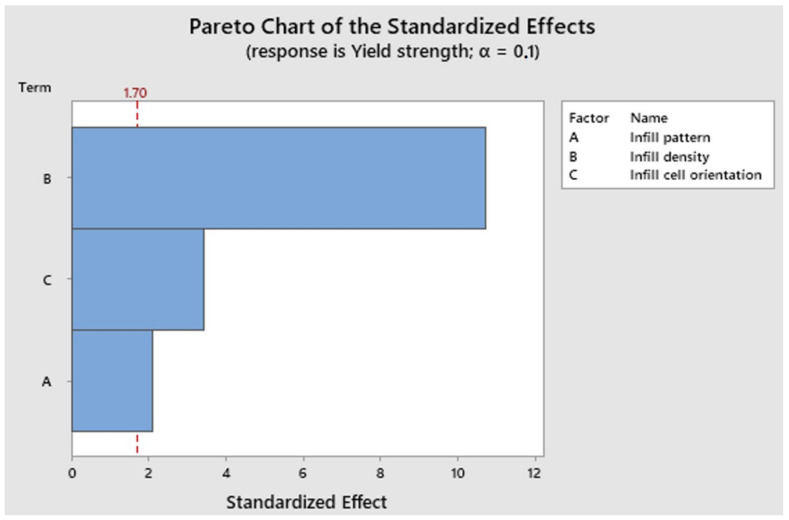
Pareto chart for the yield strength.

A Pareto chart is a statistical tool that allows us to determine the degree of impact of our studied factors on the responses. Figure 8 shows the Pareto chart for the yield strength of the tested specimens. Bars that cross the red line are considered significant so we notice that infill density is the most important factor followed by infill cell orientation and, lastly, the infill pattern.

**Figure 9:**
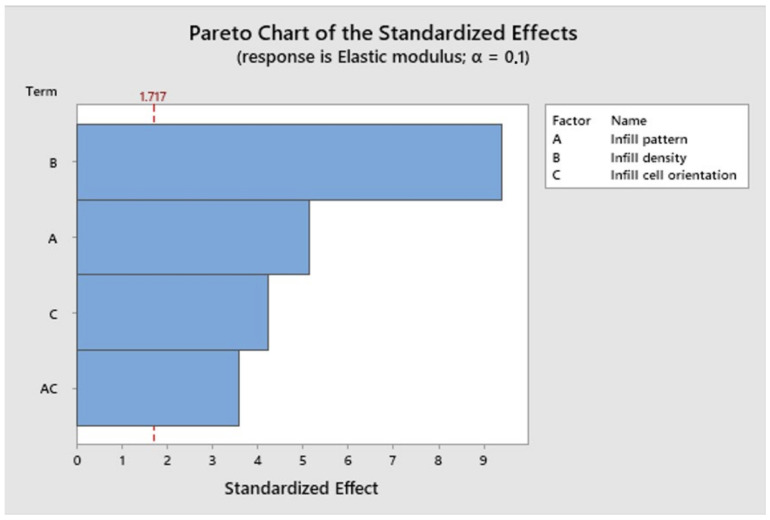
Pareto chart for the elastic modulus.

Figure 9 represents the Pareto chart for the Elastic modulus of the tested specimen, and it shows that all the studied factors and the interaction between the infill pattern and infill cell orientation had significant effects on the elastic modulus.

### 3.2. Main Effects Plots

**Figure 10:**
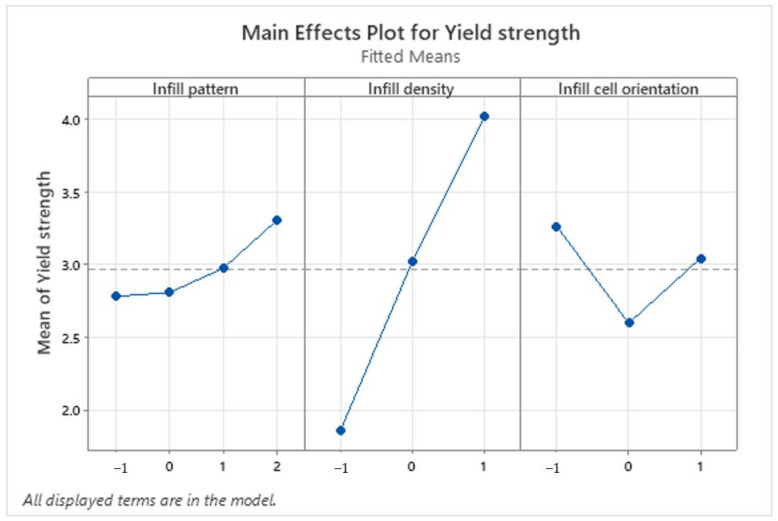
Main effects plot for the yield strength (MPA).

ANOVA analysis is used to determine the individual effect of each studied factor (infill pattern, infill density, and infill cell orientation) on the Elastic modulus and the yield strength of the tensile specimens. MINITAB2020 is the statistical software that was used to analyze the experimental data. Figure 10 represents the main effect plot for the yield strength. It is observed that the specimen that had the highest yield strength was printed with a triangle infill pattern, a 60% infill density, and an infill cell orientation of 0°, while the lowest yield strength was obtained from a printed specimen with a honeycomb infill pattern, 40% infill density and an infill cell orientation of 45°. We also noticed that the infill density had a linear effect on the yield strength.

**Figure 11:**
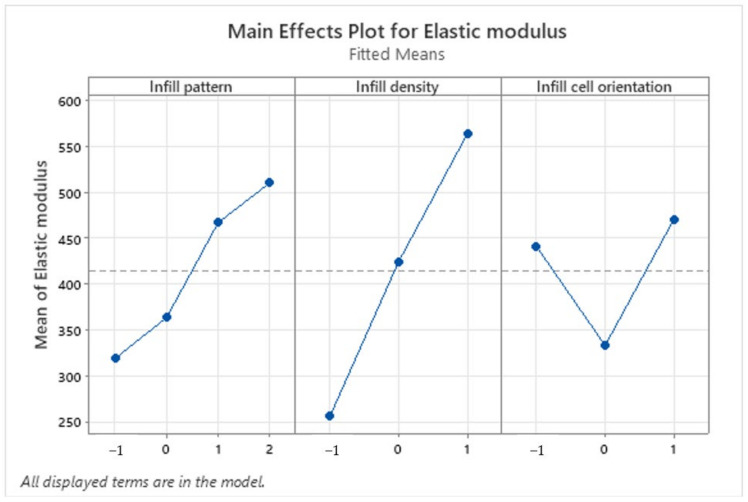
Main effects plot for the elastic modulus (MPA).

Figure 11 represents the main effect plot for the Elastic modulus. It shows that the specimen with the highest Elastic modulus was obtained with a triangle infill pattern, a 60% infill density, and an infill cell orientation of 90°. At the same time, the lowest Elastic modulus resulted from a specimen with a honeycomb infill pattern, 40% infill density, and an infill cell orientation of 45°. In addition, the effect of infill density on the Elastic modulus was also linear.

### 3.3. Interaction Plots

**Figure 12:**
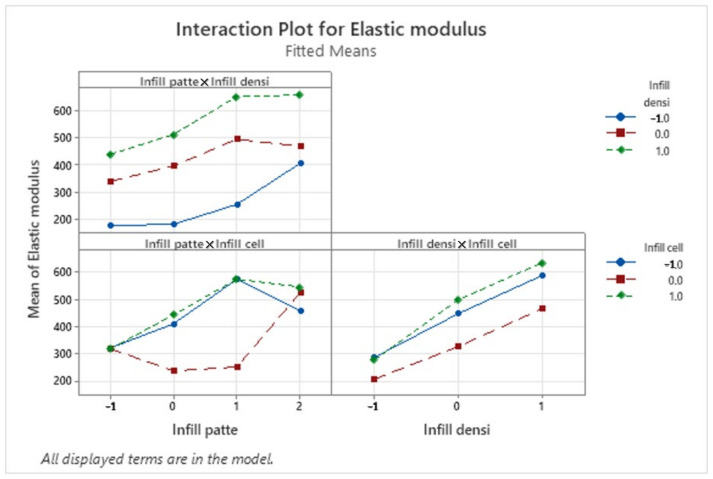
Interaction plot for the elastic modulus (MPA).

An interaction plot shows the effect of modifying one experimental factor’s settings in relation to the other factors. A two-way interaction plot shows the interaction effect between two factors. Figure 12 represents the interaction plot for the yield strength, and it shows that there was a small interaction between the infill pattern and infill cell orientation. We notice that for the four studied infill patterns, a cell orientation of 0° offers the best yield strength. The highest yield strength is obtained from printing with a triangle infill pattern, 60% infill density, and 0° infill cell orientation.

**Figure 13:**
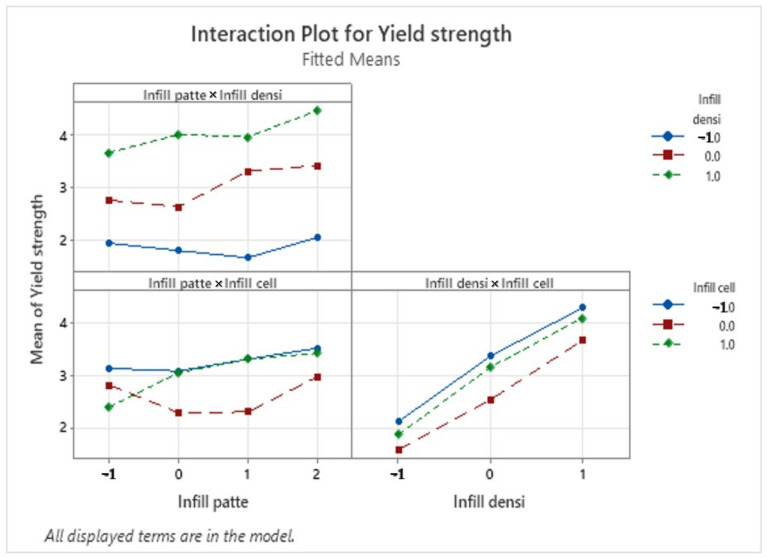
Interaction plot for the yield strength (MPA).

As for the Elastic modulus, Figure 13 shows that there is only one significant interaction was between the infill pattern and the infill cell orientation. for the 4 studied infill patterns a cell orientation of 90° offers the best Elastic modulus. The highest Elastic Modulus is obtained from a triangle infill pattern, 60% infill density and 90° infill cell orientation.

### 3.4. Regression Equation

ANOVA analysis provides a regression equation that can predict the response. Table 4 represents the model summary, and it shows that the percentage of variation explained by the model for the yield strength response is 78.51%. Equation (1) gives the regression equation of the yield strength.

(1) Yield strength=2.88+0.158×A+1.031×B−0.1744×C+0.1078×A×B+0.0982×A×C+0.014×B×C

where *A* is the infill pattern, *B* is the infill density and *C* is the infill cell orientation. This regression equation was constrained to include only the most significant coefficients identified through the Pareto chart.

**Figure 14:**
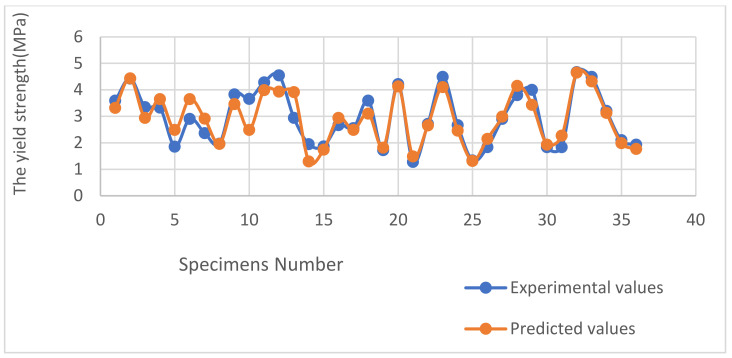
Comparison graph between the yield strength experimental and predicted values.

Figure 14 gives both the experimental results and the predicted ones by the regression Equation (1). It was noticed that the plots are close with an average error of 11.3%, which is satisfactory.

For the elastic modulus, the regression equation can explain 73.69% of the response’s variation, and it is shown in Table 5.

The following regression equation of the Elastic modulus when considering the most significant coefficients is:

(2) Elastic modulus=380.7+67.7×A+153.0×B+8.6×C+1.8×A×B+12.1×A×C+9.3×B×C

where *A* is the infill pattern, *B* is the infill density and *C* is the infill cell orientation.

**Figure 15:**
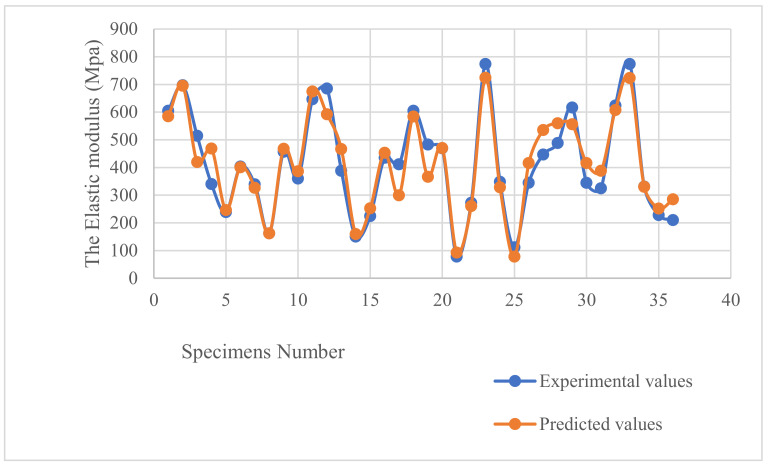
Comparison graph between the elastic modulus experimental and predicted values.

The comparison between the experimental and predicted values of the Elastic Modulus is shown in Figure 15. This figure proves that the regression equation is reliable with an average error of 11.6%.

## 4. Conclusions

In this study, our objective is to investigate the influence of relevant printing parameters on mechanical properties, namely the elastic modulus and the yielding strength. The variation of the mechanical properties is specifically related to the anisotropic aspect of the manufactured specimens with specific printing parameters, especially the raster pattern and the orientation of the printed layers relative to the loading direction. The effect of three different FDM printing parameters (the infill pattern, the infill density, and the infill cell) on the two mechanical properties, the Elastic modulus and the yield strength, were investigated using the experimental design method.

The main findings of this study were:

The most important factor is the infill density. It was observed that this factor had a linear effect on the mechanical properties. The increasing of this factor leads to the increase on the mechanical properties.

For the yield strength, it was concluded that the most important factor after the infill density is the infill cell orientation followed by the infill pattern.

For the Elastic modulus, it was found that the all studied factors had a significant effect. In addition, the interaction between the infill cell orientation and infill pattern was found to be also impactful.

The optimal levels to print parts with high yield strength are a triangle infill pattern, a 60% infill density (exceeding 60% is expected to give higher results), and an infill cell orientation of 0°.

The optimal levels to print a part with high elastic modulus are a triangle infill pattern, 60% infill density (exceeding 60% is expected to give higher results), and an infill cell orientation of 90°.

This study provides an important insight into FDM 3D printing process optimization. In a feature work we intend to study the effect of the studied process parameters on the fatigue behavior. Also, the obtained experimental results can be used to determine the anisotropy parameters of the printed parts to be considered for numerical simulations.

## Figures and Tables

**Table 1:** Default printing parameters.

Printing Parameters	
Material	Polylactic Acid (PLA)
Extrusion temperature	215 °C
Heated bed temperature	60 °C
Nozzle diameter	Ø0.4 mm
Extrusion width	0.4 mm
Top and bottom solid layer	0
Shell number	0
Printing speed	60 mm/s
Layer height	First layer is 0.3 mm and the rest are 0.2 mm

**Table 2:** FDM process parameters and their levels.

Factors	Symbols	Units	−1	0	1	2
Infill Patern	A	-----	Honeycomb	Gyroid	Grid	Triangles
Infill density	B	Percentage (%)	40	50	60	-----
Infill cell orientation	C	Degree (°)	0	45	90	-----

**Table 3:** Mixed full factorial design of experiments and tensile test results.

Infill Pattern	Infill Density	Infill Cell Orientation	Yield Strength (MPa)	Elastic Modulus (MPa)
1	0	−1	3.60 ± 0.07	605.0 ± 44
2	1	1	4.43 ± 0.08	696.5 ± 31.5
0	0	−1	3.35 ± 0.13	514.5 ± 19.5
2	0	−1	3.33 ± 0.01	339.5 ± 29
0	0	0	1.86 ± 0.16	238.5 ± 13.5
1	1	0	2.91 ± 0.19	403.0 ± 88
−1	0	1	2.37 ± 0.07	339.0 ± 9
−1	−1	−1	1.97 ± 0.12	161.5 ± 12.5
−1	1	0	3.83 ± 0.08	456.5 ± 22.5
0	1	0	3.66 ± 0.35	360.0 ± 5
2	1	0	4.29 ± 0.12	646.0 ± 5
0	1	1	4.55 ± 0.09	685.0 ± 3
−1	1	1	2.94 ± 0.12	388.0 ± 29
−1	−1	0	1.95 ± 0.04	150.0 ± 3
−1	−1	1	1.87 ± 0.03	224.0 ± 10
0	0	1	2.67 ± 0.02	434.5 ± 9
2	−1	−1	2.56 ± 0.1	411.0 ± 32
1	0	1	3.60 ± 0.07	605.0 ± 44
2	−1	0	1.73 ± 0.02	483.0 ± 95
−1	1	−1	4.22 ± 0.16	469.5 ± 18.5
1	−1	0	1.28 ± 0.04	78.0 ± 2
1	0	0	2.72 ± 0.06	272.0 ± 8
1	1	1	4.49 ± 0.1	773.5 ± 43.5
−1	0	0	2.67 ± 0.01	348.5 ± 45.5
0	−1	0	1.34 ± 0.05	111.5 ± 8.5
1	−1	−1	1.84 ± 0.09	344.5 ± 7.5
2	0	0	2.91 ± 0.01	446.5 ± 10.5
0	1	−1	3.80 ± 0.18	488.0 ± 29
2	0	1	4.00 ± 0.1	616.5 ± 16.5
1	−1	1	1.84 ± 0.09	344.5 ± 7.5
2	−1	1	1.84 ± 0.06	325.0 ± 1
2	1	−1	4.67 ± 0.14	624.0 ± 10
1	1	−1	4.49 ± 0.1	773.5 ± 43.5
−1	0	−1	3.21 ± 0.15	331.0 ± 11
0	−1	−1	2.10 ± 0.18	227.5 ± 27.5
0	−1	1	1.93 ± 0.05	210.0 ± 12

**Table 4:** The yield strength model summary.

S	R-sq	R-sq(adj)	R-sq(pred)
0.417367	87.00%	83.75%	78.51%

**Table 5:** The elastic modulus model summary.

S	R-sq	R-sq(adj)	R-sq(pred)
73.0687	90.17%	84.37%	73.69%

## Data Availability

Data are contained within the article.
